# ClpB-mediated modulation of GroEL-bound luciferase and subsequent refolding by DnaK chaperone system

**DOI:** 10.1016/j.bbrep.2026.102862

**Published:** 2026-11-03

**Authors:** Varsha, Pratiksha Pandey, Smriti Priya, Sandeep K. Sharma

**Affiliations:** a Food Toxicology Group, Food, Drug & Chemical, Environment and Systems Toxicology Division, CSIR-Indian Institute of Toxicology Research, Vishvigyan Bhawan, 31, Mahatma Gandhi Marg, Lucknow, 226001, Uttar Pradesh, India; b Academy of Scientific and Innovative Research (AcSIR), Ghaziabad, 201002, India; c System Toxicology Group, Food, Drug & Chemical, Environment and Systems Toxicology Division, CSIR-Indian Institute of Toxicology Research, Vishvigyan Bhawan, 31, Mahatma Gandhi Marg, Lucknow, 226001, Uttar Pradesh, India

**Keywords:** ClpB, DnaK, GroEL, Molecular chaperones, Protein folding, Luciferase

## Abstract

In prokaryotes, DnaK-DnaJ-GrpE (KJE), GroEL-GroES (ELS) and the AAA+ disaggregase ClpB constitute the major ATP-dependent chaperone systems involved in protein quality control machinery. Although the individual functions and interplay of KJE and ClpB systems have been extensively studied, the role of ClpB with ELS during KJE-mediated substrate refolding is still elusive. Here, we investigated the role of ClpB in KJE- and ELS-mediated refolding of chemically and heat-denatured luciferase. Simultaneous presence of KJE, ELS and ClpB significantly enhanced the refolding of chemically denatured luciferase, whereas the combination of KJE and ClpB resulted in a higher refolding yield of heat-denatured luciferase. The inhibitory effect of ELS on KJE-mediated refolding of luciferase was significantly reduced when KJE, ELS and ClpB were present simultaneously. Sequential chaperone addition experiments showed that ELS-bound heat-denatured luciferase was refolded to 24% and 36% of its native activity by KJE and ClpB respectively, whereas the KJE-ClpB chaperone system further enhanced refolding to 43%. ClpB alone did not enhanced the refolding of ELS-bound chemically denatured luciferase, while subsequent addition of KJE or KJE-ClpB restored 36% and 44% of the native activity, respectively. Turbidity assay showed that the KJE system effectively suppressed heat-induced luciferase aggregation, whereas ClpB alone exhibited no anti-aggregation activity. Collectively, these findings demonstrate that ClpB cooperates with the KJE chaperone system to enhance substrate refolding and rescue ELS-stalled substrate proteins, thus providing insights into the coordinated action of ATP-dependent chaperone systems in the maintenance of cellular protein homeostasis.

## Highlights

• ATP-dependent chaperone systems KJE, ELS and ClpB maintain cellular protein homeostasis.
• Investigated the role of ClpB in KJE-ELS-mediated refolding of unfolded and denatured luciferase.
• KJE-ELS with ClpB significantly enhances the refolding of chemically denatured luciferase.
• ClpB cooperates with KJE to enhance refolding and rescues ELS-stalled substrate proteins.

## 1 Introduction

Protein folding is a fundamental biological process that enables newly synthesised polypeptides to attain their native three-dimensional conformations required for biological functions [1], [2]. Exposure to environmental and physiological stress conditions destabilises native conformations of proteins leading to the formation of partially folded or unfolded intermediates that are susceptible to aggregation [3], [4]. The accumulation of these non-native proteins disrupts cellular protein homeostasis and leads to the progression of numerous disorders, including neurodegenerative diseases such as Parkinson's, Alzheimer's as well as metabolic, cardiovascular and amyloid-related diseases [5], [6], [7]. To ensure protein quality control, cells rely on an extensive protein homeostasis network that includes molecular chaperones, ubiquitin proteasome system and autophagy, that play crucial roles in *de novo* protein folding, refolding of misfolded proteins and clearance of irreversibly misfolded or aggregated proteins [8], [9].

Molecular chaperones, also known as heat shock proteins (HSPs), recognise the exposed hydrophobic regions of unfolded or misfolded proteins and promote their folding into native conformations without becoming part of final protein structure [10]. In prokaryotes, DnaK-DnaJ-GrpE (KJE) system and GroEL-GroES (ELS) chaperonin system represents the primary ATP-dependent protein folding machineries [11], [12]. The KJE chaperone system recognises exposed hydrophobic regions of non-native proteins and promotes folding through ATP-dependent cycles of substrate binding and release [13], [14]. KJE chaperone system actively unfolds or remodels the stable misfolded conformations and contributes to the disaggregation and recovery of stable, preaggregated substrates [15]. In contrast, the ELS chaperone system recognises non-native proteins and provides an isolated environment that facilitates productive folding [16]. In the protein folding pathway, KJE and ELS can act cooperatively but do not necessarily function as strictly sequential machineries. Buchberger et al. demonstrated that GroEL-associated non-native luciferase could be subsequently refolded by KJE, indicating that luciferase associated with GroEL remain competent for transfer back to KJE pathway for efficient folding [17]. Also, misfolded malate dehydrogenase (MDH) associated with the small heat-shock protein IbpB was reported to be transferred to KJE for ATP-dependent remodeling, that is subsequently folded into its native conformation by ELS chaperone system [18], [19]. Beyond direct substrate transfer, HtpG was shown to use ATP hydrolysis to facilitate the release of KJE-associated folding by altering the DnaK-substrate interactions and promoting the productive substrate folding [20]. In addition, recent work demonstrated the role of GroEL/ES in *de novo* protein folding where ELS binds to ribosome-associated nascent polypeptides and can coexist with trigger factor on long nascent chains, whereas, its interaction with DnaK is antagonistic [21]. Collectively, these findings suggest that chaperone networks regulate protein folding not only through the individual activities of specific chaperones but also through substrate transfer, competition and remodeling of kinetically trapped states. In addition to KJE and ELS, bacterial cells express the AAA+ ATPase ClpB, a member of the Hsp 100 family, that plays a crucial role in disaggregation and folding of aggregated proteins in cooperation with KJE in an ATP-dependent manner [22]. Unlike the KJE and ELS systems, ClpB primarily functions as a protein disaggregase by extracting polypeptides from stable protein aggregates through translocation across its central channel. The resulting folding-competent intermediates can subsequently be transferred to the KJE machinery for productive refolding [23]. ClpB functions in cooperation with the KJE system rather than as a stand-alone disaggregase. Early studies showed that ClpB alone is insufficient to reactivate aggregated luciferase [24], whereas its combination with KJE significantly enhances protein disaggregation and refolding [25]. Moreover, KJE can efficiently disaggregate smaller aggregates, while ClpB substantially enhances the disaggregation efficiency of KJE, particularly for more stable or difficult-to-solubilize aggregates [26].

Extensive studies have been done to investigate the individual functions of KJE [27], ELS [21] and ClpB [28] as well as their cooperation in protein quality control, including KJE-ELS [29], KJE-ClpB [30]. The KJE-ClpB chaperone system constitute a principal protein disaggregation machinery in bacterial system, particularly for heat stressed aggregates [31], [32], yet their role in substrate transfer and refolding in the presence of ELS either alone or in combination with KJE, is still elusive. The differential role of ClpB in modulation of substrates during KJE- or ELS-mediated refolding of chemically-denatured and heat-denatured luciferase is not yet clear.

In the present study, the individual and cooperative roles of KJE, ELS and ClpB chaperone systems during the refolding of chemically and heat-denatured luciferase has been investigated. Our results demonstrated that the simultaneous presence of KJE, ELS and ClpB enhances the refolding efficiency of chemically denatured luciferase. For heat-denatured luciferase, a higher refolding yield was observed with KJE-ClpB as compared to KJE-ELS-ClpB, suggesting substrate bound to ELS and the resulting inhibitory effect on KJE-mediated refolding of luciferase. Sequential addition of KJE and KJE-ClpB to the chemically- and heat-denatured luciferase preincubated with ELS, showed partial refolding of ELS-stalled substrates. KJE system prevents aggregation of heat-denatured luciferase, whereas ClpB alone has no anti-aggregation potential as evident by the turbidity assays. The study demonstrates that ClpB cooperates with KJE to enhance substrate refolding and rescues ELS-stalled substrate proteins, providing new insights into the coordinated action of bacterial ATP-dependent chaperone systems in the regulation of protein quality control.

## 2 Materials and methods

### 2.1 Materials

The Ni-NTA column (HisPrep FF 16/10) and DEAE Sepharose Fast Flow column (HiPrep Q FF 16/10, GE Healthcare) were procured from GE Healthcare (United States). Adenosine triphosphate (ATP, A2383), guanidine hydrochloride (GdnHCl, 50933), imidazole (56750), isopropyl β-d-1-thiogalactopyranoside (IPTG, I6758), magnesium acetate (228648), magnesium chloride (M2670), potassium acetate (236497), sodium chloride (S9888), and Trizma base (T6066) were obtained from Sigma-Aldrich Co. (St. Louis, USA). 2-Mercaptoethanol (1610710) was purchased from Bio-Rad (California, USA). An EDTA-free protease inhibitor cocktail was procured from Roche (Switzerland).

### 2.2 Methods

#### 2.2.1 Protein purification

Luciferase, DnaK, DnaJ, GrpE, GroEL, GroES and ClpB were expressed and purified as described previously [33], [34], [35]. Briefly, the luciferase, DnaK and ClpB expressing cell lysates were loaded onto a Ni-NTA affinity column pre-equilibrated with loading buffer. After washing the column, bound proteins were eluted with buffer containing NaCl or imidazole. For the purification of GrpE and DnaJ, the N-terminal His-SUMO (Smt3) tag was cleaved by incubation with ULP1 protease (2 mg ml^−1^, 500 μl). GroEL and GroES were purified by applying supernatant onto a pre-equilibrated DEAE Sepharose Fast Flow column (HiPrep Q FF 16/10, GE Healthcare) and subsequently purified through size-exclusion chromatography with a Superose™ 6 Increase 10/300 GL column (GE Healthcare).

The purity of all recombinant proteins was determined by 12% SDS-PAGE. Purified protein fractions were pooled, concentrated using a protein concentrator and stored at -80 °C. Bicinchoninic acid (BCA) assay was performed to assess the protein concentrations by using bovine serum albumin (BSA) as the standard.

#### 2.2.2 Chemical and heat denaturation of luciferase

For chemical denaturation, luciferase (35 μM) was incubated in an unfolding buffer (50 mM Tris-acetate, 15 mM magnesium acetate, 100 mM potassium acetate and 6.0 M GdnHCl, pH 7.4) for 30 min at 25 °C. Refolding was initiated by 100-fold dilution in the refolding buffer in the absence or presence of chaperone systems as indicated in the figure legends.

For heat-denaturation, native luciferase (2 μM) was incubated at 42 °C for 10 min. The heat-denatured luciferase was subsequently diluted into refolding buffer to a final concentration of 350 nM in the absence or presence of the indicated chaperone systems.

#### 2.2.3 Luciferase refolding assays

The chemically denatured luciferase was diluted 100-fold, whereas heat-denatured luciferase was diluted into refolding buffer (50 mM Tris-acetate, 15 mM magnesium acetate, and, 100 mM potassium acetate, pH 7.4) to a final luciferase concentration of 350 nM. For chaperone-mediated refolding, denatured luciferase was diluted in refolding buffer supplemented with the indicated concentrations of chaperones, DnaK (3.5 μM), DnaJ (0.7 μM), GrpE (1.4 μM), GroEL (3.5 μM), GroES (3.5 μM) and ClpB (3 μM), either individually or in combinations as detailed in the figure legends. Chaperone-mediated refolding was initiated by the addition of ATP (final concentration of 5 mM) at 25 °C and luminescence was recorded at 5-min intervals for 60 min. For activity measurement, 5 μL of each refolding reaction was transferred to a microplate well, followed by automatic dispensing of 75 μL of luciferase assay buffer [50 mM Tris-acetate (pH 7.4), 15 mM magnesium acetate and, 100 mM potassium acetate, 5 μM D-luciferin and 5 mM ATP]. Luminescence was measured immediately for 10 s, using a BioTek Cytation 5 microplate reader.

#### 2.2.4 Turbidity assay

The aggregation of luciferase was measured by monitoring the absorbance at 550 nm using a BioTek Cytation 5 microplate reader. Native luciferase (3 μM) was incubated at 37 °C for 15 h in buffer [50 mM Tris-acetate (pH 7.4), 15 mM magnesium acetate, and 100 mM potassium acetate] either alone or in the presence of indicated chaperone combinations, including DnaK (3.5 μM), DnaJ (0.7 μM), GrpE (1.4 μM), GroEL (3.5 μM), GroES (3.5 μM) and ClpB (3 μM). KJE control, ELS control and ClpB control correspond to reaction mixture lacking luciferase and were used to determine the background turbidity of the chaperone systems. Turbidity was monitored at the interval of 1 min for 15 h by measuring absorbance at 550 nM. Aggregation was quantified by comparing absorbance at 0 h and 15 h.

## 3 Results and discussion

### 3.1 ClpB cooperatively enhances KJE-mediated luciferase refolding and reduces ELS mediated inhibition

To investigate the role of ClpB in bacterial chaperone-mediated protein refolding, chemically and heat-denatured luciferase were allowed to refold with different combinations of KJE, ELS and ClpB chaperone systems. Luciferase activity was monitored for 60 min and refolding yield was expressed as a percentage of native luciferase activity. The chaperone concentrations used in the present study (DnaK:DnaJ:GrpE = 3.5:0.7:1.4 μM; 5:1:2 molar ratio) were selected based on previously established biochemical reconstitution conditions for chaperone-mediated protein folding and refolding [34], rather than to quantitatively reproduce the intracellular chaperone pool. Therefore, the absolute concentrations and stoichiometries used in our assays should be considered experimentally defined conditions for investigating chaperone cooperation and not as a direct quantitative representation of their intracellular concentrations.

For chemically denatured luciferase, spontaneous refolding yielded ∼14% of its native activity. ELS or ClpB, alone did not enhanced the refolding, demonstrating that these chaperones are unable to facilitate productive refolding of chemically denatured luciferase. The KJE chaperone system efficiently refolded ∼60% of native luciferase activity with most of the recovery occurred during the first 20-30 min, indicating the rapid recognition and processing of chemically unfolded luciferase by the DnaK chaperone cycle. Simultaneous addition of ELS with KJE reduced the refolding yield from ∼60% to 45%, corresponding to ∼15% loss of the KJE-mediated refolding species, whereas the ELS adversely affects the KJE mediated refolding of denatured luciferase (Fig. 1a–c). In the presence of ClpB, KJE significantly enhanced the refolding efficiency, from ∼60% to ∼78%, resulting in an absolute increase of ∼18% compared with KJE alone whereas ELS showed no detectable increase in refolding activity. The simultaneous presence of KJE, ELS and ClpB chaperone systems showed ∼89% refolding of its native activity, representing the maximum refolding yield. On the addition of ClpB, refolding increased by ∼44% as compared to KJE-ELS yield (∼45%), representing a two-fold improvement (Fig. 1b and c). To provide a quantitative representation of the refolding yields, the percentage recovery was converted into the corresponding concentration of refolded luciferase (nM), based on the initial substrate concentration of 350 nM. KJE-mediated refolding yielded 197 nM of the initial 350 nM luciferase. Addition of ELS reduced the refolding yield by 16.55%, corresponding to a decrease of approximately 58 nM in refolded luciferase relative to KJE alone. This decrease in refolding indicates that the presence of ELS limits the efficiency of KJE-mediated refolding of luciferase. In contrast, addition of ClpB to KJE increased the recovery from 197 to 250 nM, corresponding to an increase in 53 nM of refoldable species, are consistent with the established functional cooperation of KJE-ELS and KJE-ClpB in the remodeling and recovery of misfolded proteins [17], [36], [37]. Interestingly, when ClpB was included together with KJE and ELS, the refolding increased from 140 nM to 332 nM, corresponding to a considerable increase in 193 nM of luciferase relative to KJE-ELS mediated refolding. The substantially greater recovery in the presence of all three chaperone systems suggests the role of ClpB beyond simply facilitating KJE-mediated refolding in the chaperone network. The results demonstrate that ClpB not only enhances KJE-mediated refolding efficiency but also substantially reduces the inhibitory action of ELS chaperone system. Previous reports have demonstrated that GdnHCl can inhibit ClpB-dependent protein disaggregation and refolding [38], [39]. To determine whether the observed ClpB-dependent enhancement could be influenced by residual GdnHCl, we repeated the corresponding chemical-denaturation experiments using urea instead of GdnHCl, while maintaining the same chaperone combinations and refolding conditions. Comparable and consistent results were obtained with those observed with GdnHCl-denatured luciferase **(**Supplementary Fig. 1a–c**).**

**Fig. 1:**
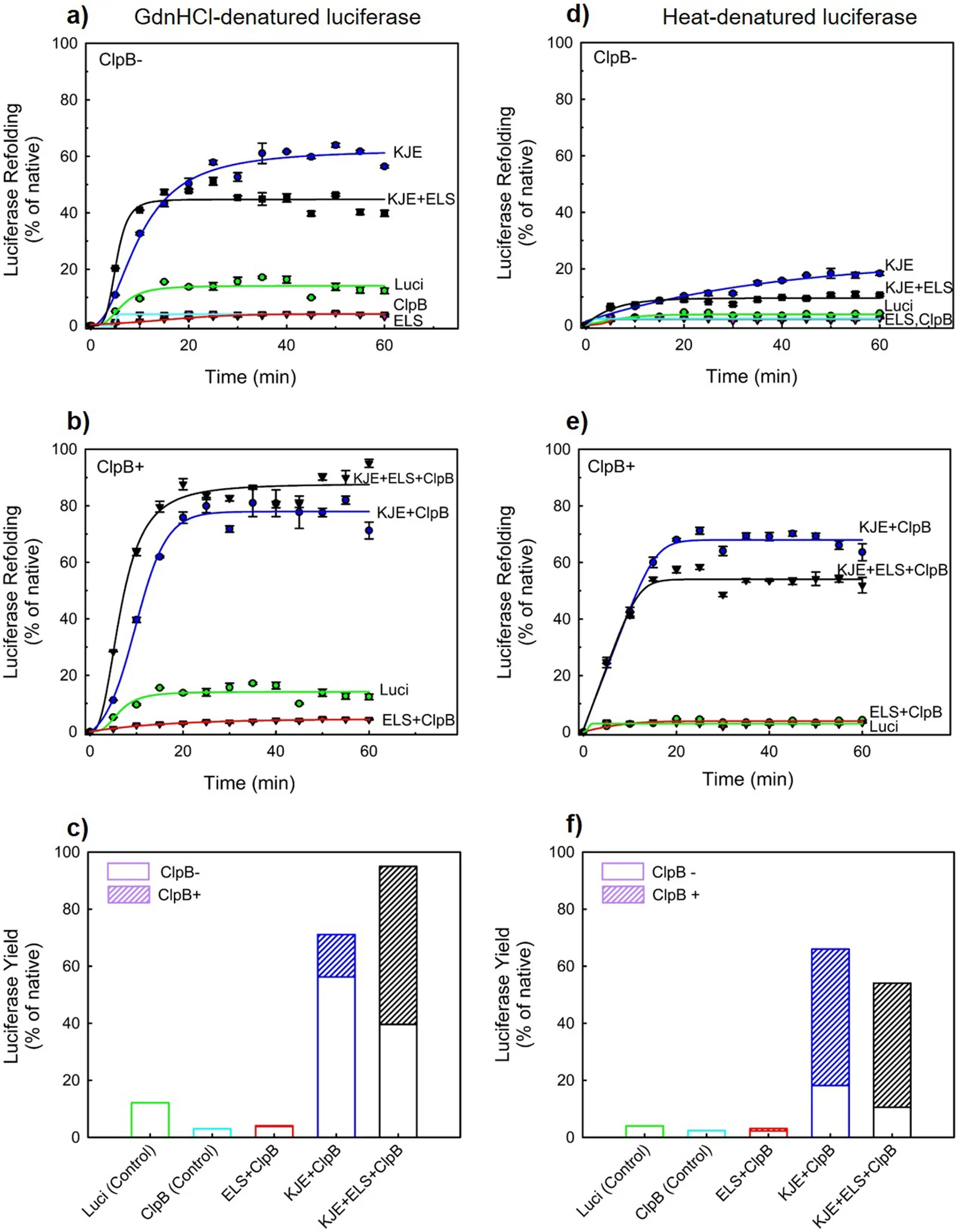
ClpB cooperatively enhances KJE-mediated luciferase refolding and reduces ELS mediated inhibition. Refolding of chemically and heat-denatured luciferase was monitored at 25 °C for 60 min, in the presence of DnaK (3.5 μM), DnaJ (0.7 μM), GrpE (1.4 μM), GroEL (3.5 μM), GroES (3.5 μM), ClpB (3 μM) and ATP (5 mM) using different combinations of chaperones (KJE, ELS, ClpB, KJE-ELS, ELS-ClpB, KJE-ClpB, KJE-ELS-ClpB. **a)** Time-dependent refolding of chemically denatured luciferase (350 nM) in the absence of ClpB. **b)** Time-dependent refolding of chemically denatured luciferase in the presence of ClpB. **c)** Net refolding yield of chemically denatured luciferase after 60 min in the absence and presence of ClpB. **d)** Time-dependent refolding of heat-denatured luciferase (350 nM) in the absence of ClpB. **e)** Time-dependent refolding of heat-denatured luciferase in the presence of ClpB. **f)** Net refolding yield of heat-denatured luciferase after 60 min in the absence and presence of ClpB. The spontaneous refolding (Luci) curves are identical in Fig. 1a and b, and in Fig. 1d and e, as the respective chemical- and heat-denaturation experiments were performed simultaneously. The activity of native luciferase (350 nM) was defined as100%. Data are presented as mean ± SEM from three independent experiments.

Heat-denatured luciferase spontaneously refolded ∼10% of its native activity, indicating that the thermal denaturation generates more aggregation-prone intermediates compared to chemically denatured substrates. Similar to chemically denatured luciferase, ELS and ClpB, individually showed no improvement in the refolding yield. The KJE chaperone system refolded ∼24% of luciferase activity, although the observed refolding was lower than that of chemically denatured luciferase suggesting the heat-induced denatured substrates are more rigid to refolding (Fig. 1d–f). The presence of ClpB with ELS did not significantly alter the refolding yield whereas with the KJE refolding yield significantly enhanced from ∼24% to ∼50%, corresponding to an increase of ∼25% and a two-fold increase compared with KJE alone. The presence of KJE-ClpB systems showed the highest refolding yield among all combinations of chaperone systems, highlighting the essential role of ClpB in the processing of heat-induced aggregates for efficient refolding. Consistent with the results observed for the chemically denatured luciferase, the simultaneous presence of KJE, ELS and ClpB chaperone systems resulted ∼42% of native luciferase activity, suggesting an absolute increase of ∼28% compared to KJE-ELS mediated refolding yield. However, unlike chemically denatured luciferase, the efficient refolding of heat denatured luciferase by the KJE-ClpB combination, suggests that the influence of ELS differs depending on the nature of unfolded substrate. The different responses of chemically and heat-denatured luciferase may be due to differences in the non-native species generated by the two treatments. Chemical denaturation largely disrupts the native interactions and produces an extensively unfolded species, whereas heat denaturation can generate a heterogeneous mixture of partially unfolded, misfolded and aggregation-prone species that retain different degrees of residual structure. The increased refolding of chemically denatured luciferase upon addition of ClpB to KJE+ELS may therefore reflect redistribution or processing of extensively unfolded species, making them more available for productive KJE-mediated refolding. In contrast, heat denaturation can generate a more heterogeneous and aggregation-prone species, for which the combined action of KJE and ClpB may be more effective in promoting recovery. Consequently, the accessibility and fate of luciferase intermediates within the KJE, ELS and ClpB chaperone systems may differ depending on the denaturation condition (Fig. 1e and f).

Taken together, these findings show that the substantial increase in refolding efficiency observed upon the addition of ClpB to KJE or KJE-ELS chaperone systems demonstrates that ClpB plays a central cooperative role in KJE-mediated refolding and modulating the ELS-stalled substrates to improve bacterial chaperone-dependent protein folding.

### 3.2 Sequential addition of KJE and ClpB partially restore the refolding of ELS bound denatured luciferase

To perform sequential chaperone addition experiments, chemically or heat denatured luciferase was first incubated with the ELS chaperone system for 30 min, followed by the addition of KJE, ClpB or KJE-ClpB combination. Luciferase activity was monitored for 60 min and expressed as the percentage of native luciferase activity (Fig. 2a).

**Fig. 2:**
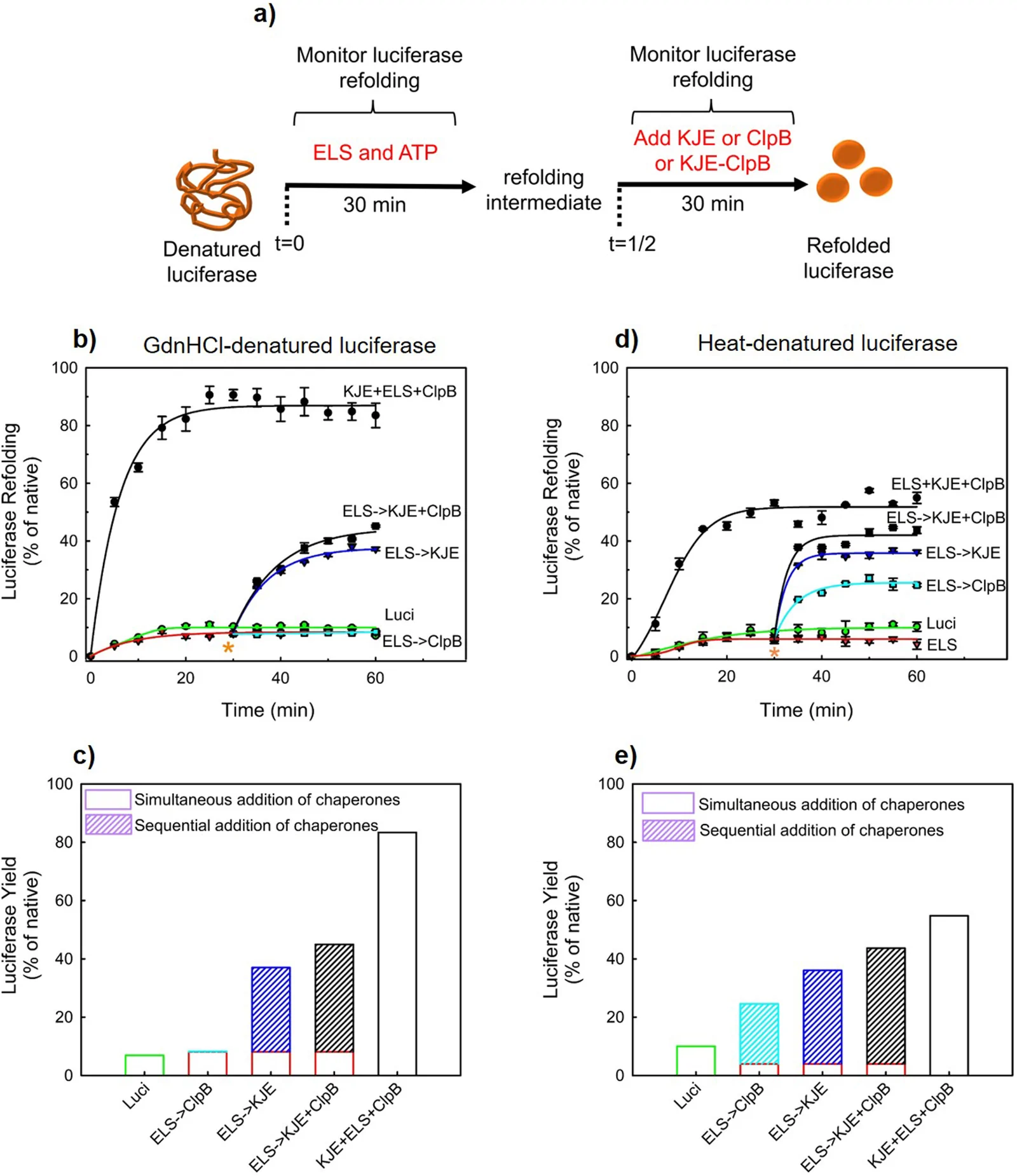
Sequential addition of KJE and ClpB partially restore the refolding of ELS bound luciferase. Refolding of chemically and heat denatured luciferase was initiated and monitored by incubation with the ELS chaperone system [GroEL (3.5 μM) and GroES (3.5 μM)] in the presence of ATP (5 mM) at 25 °C. After 30 min, KJE [DnaK (3.5 μM), DnaJ (0.7 μM), and GrpE (1.4 μM)], ClpB (3 μM), or the KJE-ClpB system was sequentially added, and luciferase refolding was monitored for an additional 30 min. **a)** Schematic representation of the sequential chaperone addition assay. **b)** Time-dependent refolding of chemically denatured luciferase following sequential addition of KJE, ClpB, or KJE-ClpB to ELS-bound substrate. **c)** Comparison of the net refolding yield of chemically denatured luciferase after simultaneous and sequential chaperone addition. **d)** Time-dependent refolding of heat-denatured luciferase following sequential addition of KJE, ClpB, or KJE-ClpB to ELS-bound substrate. **e)** Comparison of the net refolding yield of heat-denatured luciferase after simultaneous and sequential chaperone addition. The asterisk (*) indicate the time point of sequential addition of KJE, ClpB, or KJE-ClpB. The activity of native luciferase (350 nM) was defined as 100%. Data are presented as the mean ± SEM of three independent experiments.

For chemically denatured luciferase, ELS alone resulted in ∼8% of its native activity, which was even lower than the spontaneous refolding (∼10%), confirming that ELS alone inhibits the refolding. The sequential addition of KJE chaperone system significantly enhanced the refolding yield to ∼38%, an increase of ∼30% compared to ELS alone. Addition of the KJE-ClpB combination increased refolding to ∼43%, that is ∼35% and ∼13% higher as compared to ELS and KJE respectively. This demonstrates that substrates associated with the ELS chaperone system remained competent for subsequent refolding mediated by the KJE and KJE-ClpB chaperone systems. In contrast, sequential addition of ClpB alone resulted in no significant increase above the ELS baseline, demonstrating that ClpB is unable to restore the luciferase activity independently and requires functional cooperation with the KJE chaperone system for efficient refolding (Fig. 2b and c). The observations indicate that although ELS-bound luciferase remained competent for subsequent refolding, delayed addition of KJE and ClpB significantly reduced the overall refolding efficiency as compared to simultaneous addition of all three chaperone systems (Fig. 2c).

A similar trend was observed for heat-denatured luciferase (Fig. 2d and e), although the overall refolding yields were lower than those obtained with chemically denatured luciferase. The initial incubation of ELS with luciferase resulted in ∼5% of its native activity, which was lower than the spontaneous refolding yield (∼10%). The sequential addition of KJE to the pre-incubated luciferase with ELS, enhanced refolding to ∼29% of its native activity, corresponding to an increase of ∼24%. Addition of ClpB alone increased the refolding yield to ∼20% of its native activity, indicating that ClpB can partially refold heat-induced aggregates but insufficient to restore high enzymatic activity. When both KJE and ClpB chaperone systems were sequentially added together, refolding yield increased by ∼40%, highlighting that ClpB mediated disaggregation alone is insufficient to recover the enzymatic activity without the folding assistance provided by KJE chaperone system (Fig. 2d and e). Importantly, refolding obtained by sequential addition was lower than that observed with simultaneous presence of all three-chaperone systems (∼52%), indicating the early cooperation between the chaperone system is required for the optimal recovery of heat denatured substrates (Fig. 2e). To assess whether the observed ClpB-mediated enhancement extends to a GroEL-amenable substrate, we repeated the refolding experiments with malate dehydrogenase (MDH) as an additional model substrate. MDH showed preference for the GroEL/GroES (ELS) system, where ELS efficiently promoted the refolding of GdnHCl-denatured MDH. Addition of ClpB, either alone or together with KJE, resulted in a further increase in the refolding yield. However, the combined KJE+ClpB condition did not exhibit a synergistic enhancement beyond the effects observed with the individual components **(**Supplementary Fig. 2a–c**)**.

Collectively, these findings suggest that luciferase initially associated with the ELS chaperone system remained competent for refolding following the subsequent addition of KJE or KJE-ClpB.

### 3.3 KJE efficiently suppresses the thermal aggregation of luciferase

To investigate the effect of molecular chaperones on protein aggregation, the thermal aggregation of native luciferase was monitored by incubating at 37 °C for 15 h and by measuring turbidity at 550 nm. A significant increase in the turbidity was observed in luciferase alone. Comparable turbidity was observed in the presence of ELS and ClpB individually, indicate that none of these systems effectively suppressed the thermal aggregation. In contrast, the KJE chaperone system significantly suppressed luciferase aggregation throughout the incubation period, as indicated by the substantially lower absorbance at 550 nm (Fig. 3a). The addition of ClpB to ELS, KJE and ELS-KJE showed no significant changes in the turbidity indicating that KJE is primarily responsible for the suppression of thermal aggregation (Fig. 3c). Quantitative analysis of absorbance at 0 h and 15 h confirmed that samples containing the KJE chaperone system exhibited significantly lower turbidity than the samples devoid of the KJE system (Fig. 3b–d).

**Fig. 3:**
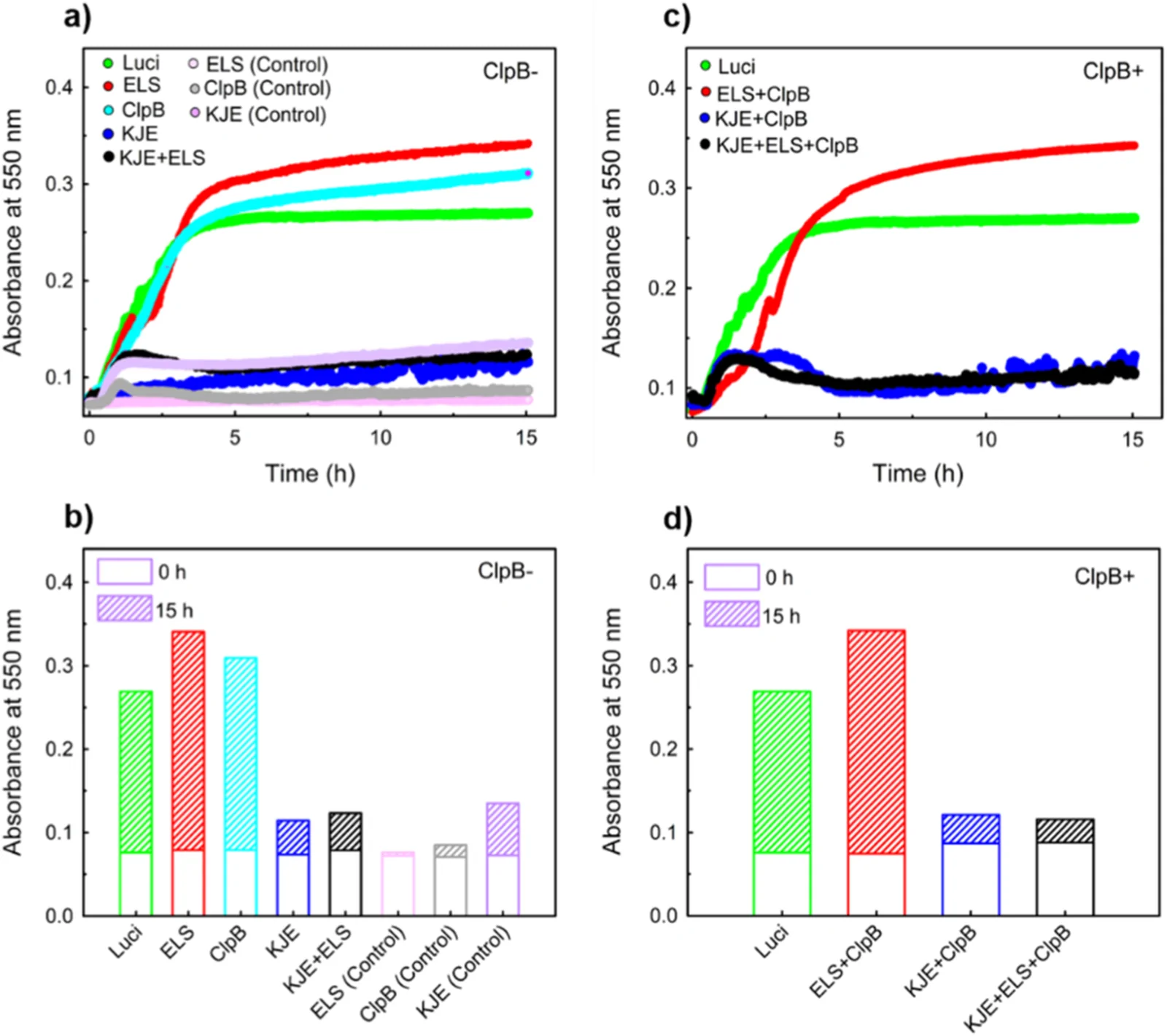
KJE suppresses the thermal aggregation of luciferase. Thermal aggregation of luciferase (3 μM) was monitored by measuring turbidity at 550 nm during incubation at 37 °C for 15 h. Luciferase was incubated alone or with DnaK (3.5 μM), DnaJ (0.7 μM), GrpE (1.4 μM), GroEL (3.5 μM), GroES (3.5 μM), ClpB (3 μM), and ATP (5 mM) in the indicated chaperone combinations. **a)** Time-dependent thermal aggregation of luciferase in the absence of ClpB. **b)** Quantitative analysis of turbidity (A550) at 0 h and 15 h in the absence of ClpB. **c)** Time-dependent thermal aggregation of luciferase in the presence of ClpB. **d)** Quantitative analysis of turbidity (A550) at 0 h and 15 h in the presence of ClpB. The turbidity curve of luciferase (Luci) are identical in Fig. 3, Fig. 1c, as the respective ClpB- and ClpB+ experiments were performed simultaneously. Data are presented as the mean ± SEM of three independent experiments.

The limited effect of ELS and ClpB, individually or in combination indicates that neither efficiently supresses thermal aggregation whereas, KJE plays a crucial role in the anti-aggregation system and acts synergistically with ClpB during protein quality control.

## 4 Conclusion

In prokaryotes, DnaK-DnaJ-GrpE (KJE), GroEL-GroES (ELS), and ClpB are major ATP-dependent chaperone systems that function individually and cooperatively to maintain protein homeostasis. The KJE system recognises exposed hydrophobic regions of non-native proteins and promotes their productive folding through ATP-dependent cycles of substrate binding and release, whereas the ELS system facilitates protein folding within an isolated chaperonin chamber. The individual activities of KJE, GroEL and ClpB, as well as the coordinated actions of the KJE-ELS and KJE-ClpB systems on non-native proteins with distinct folding properties, have been extensively studied. However, whether and how ClpB contributes to the rescue of GroEL-stalled non-native substrates remains unexplored.

The present study demonstrates a cooperative role of ClpB in modulating the interplay between ELS and KJE during luciferase refolding (Fig. 4). We observed that KJE efficiently promoted the refolding of luciferase, whereas the addition of ELS reduced KJE-mediated refolding. This reduction suggests that the association of non-native luciferase with GroEL-GroES system limits its availability for productive refolding by KJE. Such partitioning between chaperone systems may be relevant in the cellular environment, where non-native proteins can encounter multiple chaperones and may be retained within GroEL-GroES folding cycles before release or further processing.

**Fig. 4:**
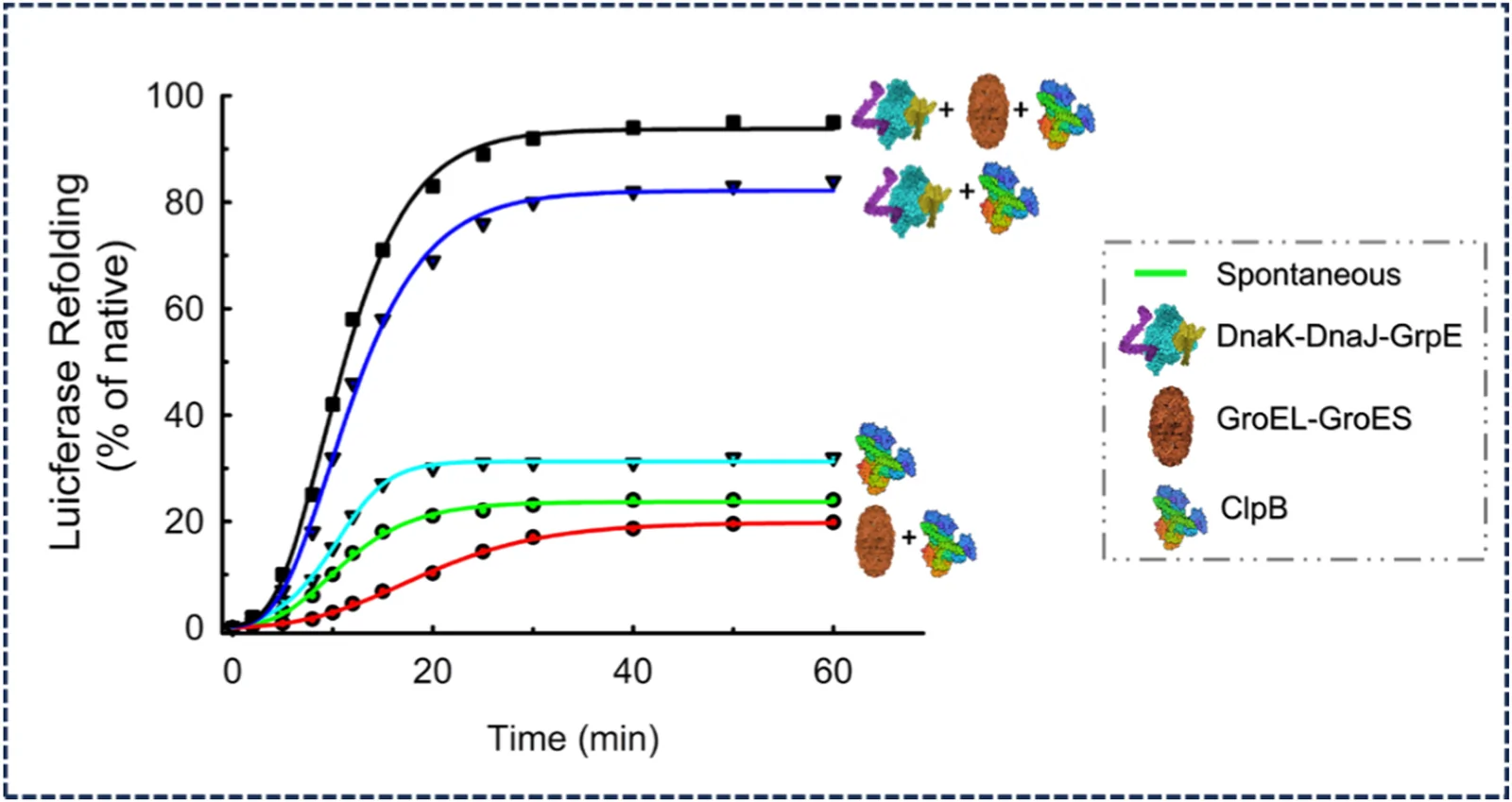
Schematic illustration of chaperone mediated refolding demonstrating the role ClpB in releasing GroEL-stalled luciferase and enhancing DnaK-mediated refolding of luciferase. During the refolding of chemically and heat denatured luciferase, ClpB alone exhibited negligible refolding efficiency, and its combination with GroEL-ES (ELS) system also did not significantly improve the refolding yield. ClpB cooperated effectively with the DnaK-DnaJ-GrpE (KJE) chaperone system, resulting in marked enhancement of luciferase refolding. The combined action of ELS, KJE, and ClpB produced the highest refolding yield, indicating that ClpB does not function as an independent folding chaperone but facilitates substrate refolding by collaborating with the KJE system, likely through the release of luciferase stalled on ELS.

The effect of ClpB was observed with both chemically and heat-denatured luciferase, although the extent and pattern of recovery differed between the two conditions. ClpB alone did not promote luciferase refolding, whereas its cooperation with KJE substantially enhanced the refolding of both chemically and heat-denatured luciferase. Notably, in the presence of KJE, ELS and ClpB, the inhibitory effect of ELS on productive luciferase refolding was markedly reduced. In the absence of ClpB, addition of ELS reduced KJE-mediated luciferase refolding, suggesting that a fraction of non-native luciferase stalled in an ELS-associated state that is less available for productive KJE-mediated refolding. However, in the presence of ClpB, the inhibitory effect of ELS was substantially reduced and the refolding yield increased markedly. Thus, the higher recovery observed in the KJE+ELS+ClpB condition does not indicate that ClpB itself enhances refolding. Rather, the results suggest that with ELS retains a fraction of luciferase in a kinetically stalled non-native state, while ClpB facilitates its redistribution or remodeling, thereby increasing its availability for productive refolding by KJE. This functional interplay of the three chaperone systems suggests that ClpB can partially overcome the inhibitory effect of ELS, thereby enabling the subsequent productive refolding of luciferase by KJE.

Sequential addition of the chaperones further showed that delayed addition of KJE or KJE+ClpB after ELS preincubation partially restored luciferase refolding, whereas simultaneous addition of the chaperones resulted in the highest refolding yield. These findings suggest that the efficiency of protein folding depends not only on the chaperone systems involved but also on their relative timing of interaction with the non-native substrate. Overall, this study provides insight into the functional interplay among KJE, ELS and ClpB and highlights the cooperation among distinct ATP-dependent chaperone systems can influence the fate of non-native proteins during protein quality control.

## CRediT authorship contribution statement

**Varsha:** Writing – original draft, Visualization, Validation, Methodology, Formal analysis, Data curation. **Pratiksha Pandey:** Writing – review & editing, Validation, Methodology, Investigation, Formal analysis, Data curation. **Smriti Priya:** Writing – review & editing, Supervision, Resources, Project administration, Funding acquisition, Conceptualization. **Sandeep K. Sharma:** Writing – review & editing, Supervision, Resources, Project administration, Funding acquisition, Conceptualization.

## Funding

The financial support was received from Indian Council of medical research, New Delhi, India under the Project ID IIRPSG-2025-01-00347. Varsha and Pratiksha Pandey received a fellowship from CSIR, India.

## Declaration of competing interest

The authors declare no conflict of interest.

## Data Availability

Data will be made available on request.

## References

[1] Kuzu O.F., Granerud L.J.T., Saatcioglu F. (2025). Navigating the landscape of protein folding and proteostasis: from molecular chaperones to therapeutic innovations. Signal Transduct. Targeted Ther..

[2] Dobson C.M. (2003). Protein folding and misfolding. Nature.

[3] Kim Y.E., Hipp M.S., Bracher A., Hayer-Hartl M., Hartl F.U. (2013). Molecular chaperone functions in protein folding and proteostasis. Annu. Rev. Biochem..

[4] Sinha A., Singh A.K., Sharma S., Trivedi R., Varsha, Kumar D., Priya S., Sharma S.K. (2025). Correlation of NADH/NAD+ electrochemical potential and enzymatic activity for investigating protein folding and kinetics. Biochem. Biophys. Res. Commun..

[5] Selkoe D.J. (2004). Cell biology of protein misfolding: the examples of Alzheimer's and Parkinson's diseases. Nat. Cell Biol..

[6] Morimoto R.I. (2008). Proteotoxic stress and inducible chaperone networks in neurodegenerative disease and aging. Genes Dev..

[7] Soto C., Pritzkow S. (2018). Protein misfolding, aggregation, and conformational strains in neurodegenerative diseases. Nat. Neurosci..

[8] Hipp M.S., Kasturi P., Hartl F.U. (2019). The proteostasis network and its decline in ageing. Nat. Rev. Mol. Cell Biol..

[9] Balch W.E., Morimoto R.I., Dillin A., Kelly J.W. (2008). Adapting proteostasis for disease intervention. Science.

[10] Hartl F.U., Bracher A., Hayer-Hartl M. (2011). Molecular chaperones in protein folding and proteostasis. Nature.

[11] Bukau B., Weissman J., Horwich A. (2006). Molecular chaperones and protein quality control. Cell.

[12] Motojima F., Yoshida M. (2015). Productive folding of a tethered protein in the chaperonin GroEL–GroES cage. Biochem. Biophys. Res. Commun..

[13] Calloni G., Chen T., Schermann S.M., Chang H.C., Genevaux P., Agostini F., Tartaglia G.G., Hayer-Hartl M., Hartl F.U. (2012). DnaK functions as a central hub in the E. coli chaperone network. Cell Rep..

[14] Hammack L.J., Firestone K., Chang W., Kusmierczyk A.R. (2017). Molecular chaperones of the Hsp70 family assist in the assembly of 20S proteasomes. Biochem. Biophys. Res. Commun..

[15] Weibezahn J., Schlieker C., Bukau B., Mogk A. (2003). Characterization of a trap mutant of the AAA+ chaperone ClpB. J. Biol. Chem..

[16] Tyagi N.K., Fenton W.A., Horwich A.L. (2009). GroEL/GroES cycling: ATP binds to an open ring before substrate protein favoring protein binding and production of the native state. Proc. Natl. Acad. Sci. U. S. A..

[17] Buchberger A., Schroder H., Hesterkamp T., Schonfeld H.J., Bukau B. (1996). Substrate shuttling between the DnaK and GroEL systems indicates a chaperone network promoting protein folding. J. Mol. Biol..

[18] Lethanh H., Neubauer P., Hoffmann F. (2005). The small heat-shock proteins IbpA and IbpB reduce the stress load of recombinant Escherichia coli and delay degradation of inclusion bodies. Microb. Cell Fact..

[19] Veinger L., Diamant S., Buchner J., Goloubinoff P. (1998). The small heat-shock protein IbpB from Escherichia coli stabilizes stress-denatured proteins for subsequent refolding by a multichaperone network. J. Biol. Chem..

[20] Marzano N.R., Skewes B., McMahon S., Rice L., Cox D., van Oijen A.M., Ecroyd H. (2026). Direct single-molecule visualization of Hsp90-mediated relief of an Hsp70-folding block. Sci. Adv..

[21] Roeselová A., Maslen S.L., He J.Z., Jurkeviciute G., Pajak A., Skehel J.M., Enchev R.I., Balchin D. (2025). GroEL/ES chaperonin unfolds then encapsulates a nascent protein on the ribosome. Nat. Commun..

[22] Mogk A., Kummer E., Bukau B. (2015). Cooperation of Hsp70 and Hsp100 chaperone machines in protein disaggregation. Front. Mol. Biosci..

[23] Haslberger T., Zdanowicz A., Brand I., Kirstein J., Turgay K., Mogk A., Bukau B. (2008). Protein disaggregation by the AAA+ chaperone ClpB involves partial threading of looped polypeptide segments. Nat. Struct. Mol. Biol..

[24] Zolkiewski M. (1999). ClpB cooperates with DnaK, DnaJ, and GrpE in suppressing protein aggregation. A novel multi-chaperone system from Escherichia coli. J. Biol. Chem..

[25] Doyle S.M., Shastry S., Kravats A.N., Shih Y.H., Miot M., Hoskins J.R., Stan G., Wickner S. (2015). Interplay between E. coli DnaK, ClpB and GrpE during protein disaggregation. J. Mol. Biol..

[26] Diamant S., Ben-Zvi A.P., Bukau B., Goloubinoff P. (2000). Size-dependent disaggregation of stable protein aggregates by the DnaK chaperone machinery. J. Biol. Chem..

[27] Chiappori F., Fumian M., Milanesi L., Merelli I. (2015). DnaK as antibiotic target: hot spot residues analysis for differential inhibition of the bacterial protein in comparison with the human HSP70. PLoS One.

[28] Guenther I., Zolkiewski M., Kędzierska-Mieszkowska S. (2012). Cooperation between two ClpB isoforms enhances the recovery of the recombinant β-galactosidase from inclusion bodies. Biochem. Biophys. Res. Commun..

[29] Langer T., Lu C., Echols H., Flanagan J., Hayer M.K., Hartl F.U. (1992). Successive action of DnaK, DnaJ and GroEL along the pathway of chaperone-mediated protein folding. Nature.

[30] Hoskins J.R., Wickramaratne A.C., Jewell C.P., Jenkins L.M., Wickner S. (2025). Hsp90, DnaK, and ClpB collaborate in protein reactivation. Proc. Natl. Acad. Sci..

[31] Ngansop F., Li H., Zolkiewska A., Zolkiewski M. (2013). Biochemical characterization of the apicoplast-targeted AAA+ ATPase ClpB from Plasmodium falciparum. Biochem. Biophys. Res. Commun..

[32] Goloubinoff P., Mogk A., Zvi A.P., Tomoyasu T., Bukau B. (1999). Sequential mechanism of solubilization and refolding of stable protein aggregates by a bichaperone network. Proc. Natl. Acad. Sci. U. S. A..

[33] Xiao X., Fay A., Molina P.S., Kovach A., Glickman M.S., Li H. (2024). Structure of the M. tuberculosis DnaK−GrpE complex reveals how key DnaK roles are controlled. Nat. Commun..

[34] Jacobson T., Priya S., Sharma S.K., Andersson S., Jakobsson S., Tanghe R., Ashouri A., Rauch S., Goloubinoff P., Christen P., Tamás M.J. (2017). Cadmium causes misfolding and aggregation of cytosolic proteins in yeast. Mol. Cell Biol..

[35] Seyffer F., Kummer E., Oguchi Y., Winkler J., Kumar M., Zahn R., Sourjik V., Bukau B., Mogk A. (2012). Hsp70 proteins bind Hsp100 regulatory M domains to activate AAA+ disaggregase at aggregate surfaces. Nat. Struct. Mol. Biol..

[36] Glover J.R., Lindquist S. (1998). Hsp104, Hsp70, and Hsp40: a novel chaperone system that rescues previously aggregated proteins. Cell.

[37] Schlieker C., Weibezahn J., Patzelt H., Tessarz P., Strub C., Zeth K., Erbse A., Schneider-Mergener J., Chin J.W., Schultz P.G., Bukau B., Mogk A. (2004). Substrate recognition by the AAA+ chaperone ClpB. Nat. Struct. Mol. Biol..

[38] Kummer E., Oguchi Y., Seyffer F., Bukau B., Mogk A. (2013). Mechanism of Hsp104/ClpB inhibition by prion curing guanidinium hydrochloride. FEBS (Fed. Eur. Biochem. Soc.) Lett..

[39] Nowicki Ł., Leźnicki P., Morawiec E., Litwińczuk N., Liberek K. (2012). Role of a conserved aspartic acid in nucleotide binding domain 1 (NBD1) of Hsp100 chaperones in their activities. Cell Stress Chaperones.

