# Supplementary material for: ClpB-mediated modulation of GroEL-bound luciferase and subsequent refolding by DnaK chaperone system
Source: Biochem Biophys Rep. 2026 Nov 3;48:102862. doi: 10.1016/j.bbrep.2026.102862 (PMC13756050; doi:10.1016/j.bbrep.2026.102862)
Supplement: Supplementary file 1 [file mmc1.docx]

**Supplementary Information for**

**ClpB-mediated modulation of GroEL-bound Luciferase and subsequent refolding by DnaK Chaperone System**

Varsha^1,2^, Pratiksha Pandey^1,2^, Smriti Priya^2,3*^, Sandeep K. Sharma^1,2*^

^1^Food Toxicology Group, Food, Drug & Chemical, Environment and Systems Toxicology Division, CSIR-Indian Institute of Toxicology Research, Vishvigyan Bhawan, 31, Mahatma Gandhi Marg, Lucknow-226001, Uttar Pradesh, India.

^2^Academy of Scientific and Innovative Research (AcSIR), Ghaziabad, 201002, India.

^3^System Toxicology Group, Food, Drug & Chemical, Environment and Systems Toxicology Division, CSIR-Indian Institute of Toxicology Research, Vishvigyan Bhawan, 31, Mahatma Gandhi Marg, Lucknow-226001, Uttar Pradesh, India.

***Corresponding Author**

Dr. Sandeep K. Sharma

CSIR-Indian Institute of Toxicology Research,

Vishvigyan Bhawan, 31, Mahatma Gandhi Marg,

Lucknow-226001, Uttar Pradesh, India

E-mail: [sandeep.sharma@csir.res.in](mailto:sandeep.sharma@iitr.res.in)

Phone: +91-522-2217497-767

Dr. Smriti Priya

CSIR-Indian Institute of Toxicology Research,

Vishvigyan Bhawan, 31, Mahatma Gandhi Marg,

Lucknow-226001, Uttar Pradesh, India

E-mail:  smriti.priya@csir.res.in

Phone: +91-522-2217497-65

**This file includes:**

Supplementary Figure S1…………………………………………………………..…….........3

Supplementary Figure S2…………………………………………………………..…….........4

Supplementary Figure S4…………………………………………………………..…….........5

Supplementary Figure S5…………………………………………………………..……........6

**Supplementary fig:1. ClpB cooperatively enhances KJE-mediated luciferase refolding and reduces ELS mediated inhibition.** Refolding of urea-denatured luciferase was monitored at 25 ºC for 60 min, in the presence of DnaK (3.5 μM), DnaJ (0.7 μM), GrpE (1.4 μM), GroEL (3.5 μM), GroES (3.5 μM), ClpB (3 μM) and ATP (5 mM) using different combinations of chaperones (KJE, ELS, ClpB, KJE-ELS, ELS-ClpB, KJE-ClpB, KJE-ELS-ClpB. **a)** Time-dependent refolding of chemically denatured luciferase (350 nM) in the absence of ClpB. **b)** Time-dependent refolding of chemically denatured luciferase in the presence of ClpB. **c)** Time-dependent refolding of urea denatured luciferase following sequential addition of KJE, ClpB, or KJE-ClpB to ELS-bound substrate.

**Supplementary fig:2. Refolding of GnHCl denatured MDH.** Refolding of GnHCl-denatured MDH (700 nM) was monitored at 25 ºC for 120 min, in the presence of DnaK (3.5 μM), DnaJ (0.7 μM), GrpE (1.4 μM), GroEL (3.5 μM), GroES (3.5 μM), ClpB (3 μM) and ATP (5 mM) using different combinations of chaperones (KJE, ELS, ClpB, KJE-ELS, ELS-ClpB, KJE-ClpB, KJE-ELS-ClpB. **a)** Time-dependent refolding of chemically denatured MDH (700 nM) in the absence of ClpB. **b)** Time-dependent refolding of chemically denatured MDH (700 nM) in the presence of ClpB. **c)** Time-dependent refolding of chemically denatured MDH following sequential addition of KJE, ClpB, or KJE-ClpB to ELS-bound substrate.

**Supplementary fig:3. Effect of ClpB on KJE- and ELS-mediated refolding of chemically and heat-denatured luciferase.** (a, c) Simultaneous addition of the indicated chaperones to chemically and heat-denatured luciferase, respectively. (b, d) Sequential addition experiments in which luciferase was first incubated with ELS before addition of KJE, ClpB, or KJE+ClpB. Refolding was measured by luciferase activity and represented relative to native luciferase activity (350 nM). Data represent mean ± SEM from three independent experiments; individual points indicate independent experiments. Statistical analysis was performed using one-way ANOVA.

**Supplementary fig:4. Time-dependent refolding of heat-denatured luciferase following incubation with ELS.** Heat-denatured luciferase was incubated with GroEL (3.5 μM) and GroES (3.5 μM) for 30 min, followed by addition of ClpB (3 μM), MDH (3 μM), BSA (3 μM), or Ficoll (3 μM). ATP (5 mM) was present throughout the reaction. Ficoll was included as a control to assess potential nonspecific/macromolecular effects. Luciferase activity was monitored over time and expressed as the percentage of native activity.
